# Homograft Versus Valves and Valved Conduits for Extensive Aortic Valve Endocarditis with Aortic Root Involvement/Destruction: A Systematic Review and Meta-Analysis

**DOI:** 10.1055/s-0042-1743110

**Published:** 2022-08-07

**Authors:** Michael L. Williams, John D. L. Brookes, Joseph S. Jaya, Eren Tan

**Affiliations:** 1 Department of Cardiothoracic Surgery, Royal Prince Alfred Hospital, Sydney, New South Wales, Australia; 2 Department of Surgery, Monash Health, Victoria, Australia; 3 Department of Surgery, Eastern Health, Victoria, Australia

**Keywords:** endocarditis, aortic valve, homograft, aortic root abscess

## Abstract

Aortic valve infective endocarditis is a life-threatening condition. Patients frequently present profoundly unwell and extensive surgery may be required to correct the underlying anatomical deficits and control sepsis. Periannular involvement occurs in more than 10% of patients with aortic valve endocarditis. Complex aortic valve endocarditis has a mortality rate of 10 to 40%. Longstanding surgical dogma suggests homografts represent the optimal replacement option in complex aortic valve endocarditis; however, there is a paucity of evidence and lack of consensus on the optimal replacement choice. A systematic review and meta-analysis was performed utilizing EMBASE, PubMed, and the Cochrane databases to review articles describing homografts versus aortic valve replacement and/or valved conduit graft implantation for complex aortic valve endocarditis. The outcomes of interest were mortality, reinfection, and reoperation. Eleven studies were included in this meta-analysis, contributing 810 episodes of complex aortic valve endocarditis. All included reports were cohort studies. There was no statistically significant difference in overall mortality (risk ratio [RR] 0.99; 95% confidence interval [CI], 0.61–1.59; *p* = 0.95), reinfection (RR 0.89; 95% CI, 0.45–1.78; *p* = 0.74), or reoperation (RR 0.91; 95% CI, 0.38–2.14; *p* = 0.87) between the homograft and valve replacement/valved conduit graft groups. Overall, there was no difference in mortality, reinfection, or reoperation rates between homografts and other valve or valved conduits in management of complex aortic endocarditis. However, there is a paucity of high-quality evidence in the area, and comparison of valve types warrants further investigation.

## Introduction

Complex infection of the aortic valve with aortic root involvement/destruction remains a grave challenge to the cardiothoracic surgeon. Active infective endocarditis is a life-threatening condition, and periannular involvement occurs in more than 10% of patients with aortic valve endocarditis. 1 2 3 Patients frequently present profoundly unwell and extensive surgery may be required to correct the underlying anatomical deficits and provide source control in septic patients. Complex aortic valve endocarditis with aortic root involvement continues to have a high perioperative mortality between 10 and 40%. 3 4 5

However, the optimal surgical approach remains an area of controversy. Longstanding surgical dogma has dictated that homografts should be used to minimize the amount of prosthetic material left in an infected operative bed. However, recent studies have supported the use of conventional mechanical or bioprosthetic valves and valved conduit grafts in the setting of aortic annular disruption, fistula, abscess, and prosthetic valve endocarditis. 6 7 8 9 10

As noted by Kim and Sundt, 11 limitations in the available evidence necessitate the application of considerable degrees of judgment and nuance of opinion expressed in the creation of guidelines. Keeping this statement in mind, the Society of Thoracic Surgeons (STS) guidelines are outlined in Table 1 .

**Table 1:** Society of Thoracic Surgeons complex aortic root infective endocarditis recommendations

Society of Thoracic Surgeons guidelines	Level of evidence
When periannular abscess is associated with infective endocarditis, it is reasonable to use a mechanical or stented tissue valve if radical debridement is performed and the valve can be anchored to healthy and strong tissue	(Class IIa, Level of evidence B)
Aortic homografts are considered reasonable for native aortic valve endocarditis particularly with periannular abscess and extensive annular or aortic wall destruction requiring aortic root replacement/reconstruction of extensive aortic-ventricular discontinuity	(Class IIb, Level of evidence B)
In the setting of prosthetic valve endocarditis (PVE) with periannular abscess a homograft can be beneficial in aortic PVE when periannular abscess or extensive ventricular-aortic discontinuity is present, or when aortic root replacement/reconstruction is necessary because of annular destruction or destruction of anatomical structures	(Class IIa, Level of evidence B)

Note: Class II indicates that there is: Conflicting evidence and/ or a divergence of opinion about the usefulness/efficacy of a procedure; IIa: Weight of evidence/opinion is in favor of efficacy; IIb: Usefulness/efficacy is less well established by evidence/opinion. 35 36

Management of complex infective endocarditis incorporates several key surgical tenets including: (1) debridement of all infected tissue, (2) excluding the abscess cavity from the circulation, (3) restoration of aorto-ventricular continuity, (4) reconstruction and replacement of the debrided structures, (5) allowing safe anchorage for valvular implants, and (6) targeted antibiotic therapy to prevent recurrence of infection. 12 13 14

Many have accepted the aortic homograft as best fulfilling these tenets to correct complex aortic valve endocarditis. Homografts provide the possibility to broadly resect areas of infection, particularly toward the anterior mitral leaflet, while making use of the homograft to patch any residual defects. Additionally, by decreasing prosthetic tissue in the postoperative bed, aortic homografts have been felt to suffer lower rates of reinfection in the setting of complex aortic valve endocarditis. 9 15

However, there are several challenges confronting the use of homografts. In many centers homografts are not readily available, or have only recently become available. The storage, harvest, and distribution of homografts remains expensive and logistically difficult compared with the use of readily available prosthetic valves. Indeed, for those unfamiliar with or for those who do not routinely use homografts it may not be the simplest solution. Furthermore, there are reports of early calcification and degeneration of the homograft. 4 16 17 18

In light of these limitations and recent studies increasingly emphasizing the role of conventional valves and valved conduit grafts, this meta-analysis sought to address those deficiencies of evidence highlighted by Kim and Sundt. 11 This article sought to assess whether the use of homografts compared with conventional valves and valved conduit grafts reduces high patient mortality, need for high-risk reoperation, and rates of reinfection in the setting of complex aortic valve endocarditis with aortic root involvement/destruction. The primary outcome of interest was overall mortality in homografts compared with valves and valved conduits. Secondary outcomes were mortality by valve and valved conduit type (tissue vs. mechanical), and rates of reinfection and reoperation.

## Methods

### Literature Search Strategy

A systematic review and meta-analysis was undertaken in keeping with the QUOROM guidelines. 19 PubMed, EMBASE, and Cochrane electronic databases were used to perform a structured search. The databases were searched for articles concerning the treatment of complex aortic valve endocarditis with aortic root involvement/destruction that were published from January 1990 to June 2019. The search strategy included a combination of keywords including “aortic root abscess,” “periannular abscess,” “aortic valve endocarditis,” “homograft,” “aortic valve replacement,” and “aortic root endocarditis.”

### Selection Criteria

Studies were included that provided primary data from randomized control trials or cohort studies directly comparing homografts to valve replacements and/or valved conduit grafts for complex infective aortic valve endocarditis. Complex aortic valve endocarditis was defined as presence of aortic root abscess, gross annular disruption, aorto-ventricular discontinuity, fistula, etc. To be eligible for inclusion, studies had to report specific outcomes (rates of mortality, reinfection, and reoperation) for valve/conduit graft type, and had to clearly delineate outcomes for complex aortic valve endocarditis (rather than uncomplicated aortic valve endocarditis). Reoperation was defined in terms of whether the patients had further valvular or aortic operations within the cohort of interest. Reinfection was defined as further episodes of infective endocarditis with the same or different organism as the primary infection. Minimum cohort size for inclusion in this systematic review was 10 patients. Included studies were limited to those in English language and only involving human subjects. Abstracts, reviews, correspondences, editorials, or conference proceedings were excluded from further analysis.

### Data Extraction and Critical Appraisal

After removing duplicates, articles were screened by title and abstract for eligibility. All retrieved articles were independently reviewed by two separate reviewers (J.B. and J.J.). Subsequently, eligible articles then underwent full-text review if they met inclusion criteria. In case of discrepant opinions decisions were made following group discussion and consensus between the authors. Data was extracted from eligible studies regarding patient characteristics into a standard form (number, gender, mean age, history of previous operation, valve conduits, microbiological culture results, and clinical outcomes—rates of mortality, reinfection, and reoperation). Clinical outcomes were extracted based upon reported inpatient, 1-, 5-, and 10-year results. The methodological quality of the selected articles was assessed using the Newcastle–Ottawa Scale for assessment of cohort studies. 20

### Statistical Analysis

A meta-analysis was performed using Rev Man 5.0 (Review Manager Version 5.3, The Nordic Cochrane Centre, The Cochrane Collaboration, Copenhagen, Denmark). 21 Random-effects model was employed to allow for the heterogeneity of the cohorts within the included studies. The effect measures estimated were risk ratio (RR) for dichotomous data that was reported with 95% confidence intervals (CIs). RR was chosen for clarity of results, as it is the most readily understood comparitory statistic. 22 A RR of less than 1 favored the homograft group. Heterogeneity was assessed using the *I* ^2^ statistic. Statistical significance was determined at a *p* -value of 0.05.

## Results

### Quality of Studies

**Fig. 1:**
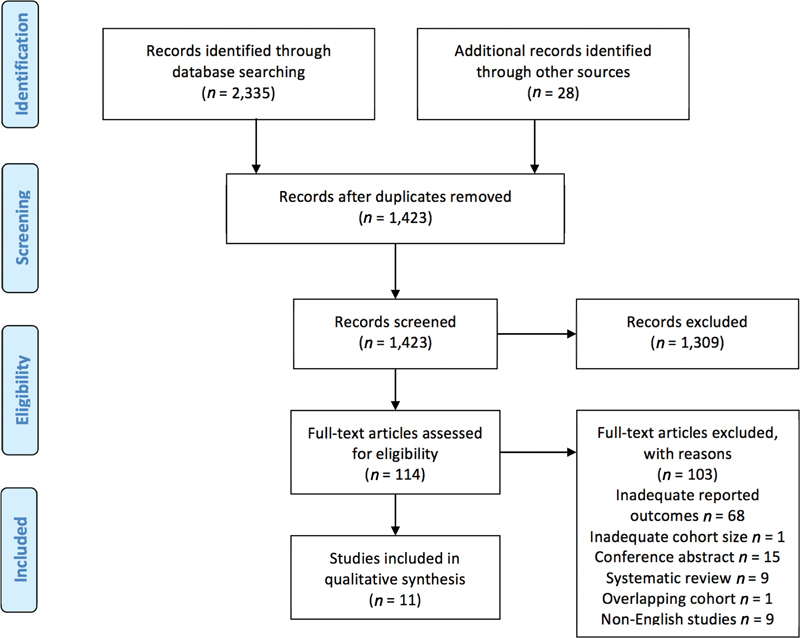
Preferred Reporting Items for Systematic Reviews and Meta-Analyses (PRISMA) flowchart summarizing the search strategy for relevant publications.

The literature search identified a total of 2,335 studies ( Fig. 1 ). Once duplicates were removed this left 1,423 articles. After exclusion of irrelevant studies by screening titles and abstracts, 114 records were selected to undergo full-text review. A manual hand search was performed from the reference lists of relevant articles that underwent full-text review and this yielded a further potential 28 studies that were similarly screened by title and abstract for eligibility for full-text review.

Studies were screened for duplication of study or cohort by authors and institutions. This revealed three studies appropriate for inclusion (Siniawski et al, 2003, Knosalla et al, Siniawski et al, 2005) to have come from the same institution. 9 23 24 The decision was made to include Siniawski et al (2003) and Knosalla et al, and exclude Siniawski et al (2005). Siniawski et al (2005) looked specifically at those patients who also required mitral valve operations in addition to the operation for their aortic valve endocarditis. Therefore, this was a smaller and more specific subset than those available from the other two studies deemed eligible for inclusion and this patient population also overlapped with those included in the other two studies. Knosalla et al (patients operated 1988–1996) and Siniawski et al (2003) (patients operated 1997–2001) did not overlap in their patient population. One other study by Lupinetti and Lemmer, comprised of only 5 patients and so was therefore excluded from further analysis for not meeting the minimum 10-patient inclusion criteria. 25

This left 11 studies that met the inclusion criteria to undergo quantitative analysis ( Table 2 ). 4 7 8 9 10 23 26 27 28 29 30 The 11 studies yielded 810 episodes of complex aortic valve infective endocarditis. All of these episodes were complicated by aortic root abscess, infected pseudoaneurysm, fistula, annular disruption, or other forms of aorto-ventricular discontinuity secondary to infection.

**Table 2:** Study characteristics of those included in quantitative meta-analysis

Author, Institution, Country	Year	Number contributed		Years of enrolment	Purely complex aortic	Mortality rate	ReInf rate	Reop rate	Age (mean)	Duration F-up/ completeness of F-up	Gender (male)
Elgalad, 26 Tanta University, Egypt	2019	H = 24	Other valve and graft 144	2000–2013	All complex 44.6% PVE ( *n* = 75)	28.0% ( *n* = 47)	13.7% ( *n* = 23)	NR	69.5 years	Median 4.5 years (1 day–13.25 years) No comment loss to F-up	32.7% ( *n* = 55)
Ramos, 27 Hospital Universitario Puerta de Hierro, Spain	2016	H = 2	AVR = 6, AVC = 8	2008–2015	SC 16 100% PVE ( *n* = 16)	18.75% ( *n* = 3)	0.0% ( *n* = 0)	NR	56.1 years	NR	93.8% ( *n* = 15)
Lee, 4 Yonsei University College of Medicine, South Korea	2014	H = 3	mAVR = 38, tAVR = 7	1999–2012	All complex 41.7% PVE ( *n* = 20)	12.5% ( *n* = 6)	0.0% ( *n* = 0)	2.1% ( *n* = 1)	50 years	68.7 months (2–159 months) 97.3% complete follow-up in survivors	75% ( *n* = 36)
Jassar, 7 University of Pennsylvania Medical Centre, USA	2012	H = 36	mAVC = 43, tAVC = 55	2000–2010	All complex 67.2% PVE ( *n* = 90)	40.3% ( *n* = 54)	10.4% ( *n* = 14)	4.5% ( *n* = 6)	58.3 years	Mean 32.1 months 80.8% of survivors	70.9% ( *n* = 95)
Avierinos, 28 Hopital Timone, France	2007	H = 41	tAVR = 15, mAVR = 7	1990–2005	SC 63 36.5% PVE ( *n* = 23/63)	11.1% ( *n* = 7)	12.7% ( *n* = 8)	3.2% ( *n* = 1)	57 years total cohort NR Complex aortic root	Did not report for complex patients 98% completeness follow-up Median 2 years, maximum 12 years for total study	87% total cohort Did not report for complex
David, 8 University of Toronto, Canada	2007	H = 14	mAVR = 66, tAVR = 55	1978–2004	All complex 48.9% PVE ( *n* = 66)	15.6% ( *n* = 21)	NRVT	NRVT	51 years	6.2 years (range 0–22 years) Complete F-up 100%	68% ( *n* = 92)
Leyh, 10 Hannover Medical School, Germany	2004	H = 16	mAVC = 13	1991–2001	All complex 100% PVE ( *n* = 29)	17.2% ( *n* = 5)	0.0% ( *n* = 0)	0.0% ( *n* = 0)	55.2 years	Range 1–152 months Complete F-up 100%	79.3% ( *n* = 23)
Siniawski, 23 German Heart Institute, Germany	2003	H = 68	tAVC = 25	1997–2001	All complex 41.9% PVE ( *n* = 39)	14.0% ( *n* = 13)	5.4% ( *n* = 5)	NR	51.9 years	NR	66.7% ( *n* = 62)
Gulbins, 29 University Hospital Grosshadern, Germany	2002	H = 24	mAVR = 7		SC 31 PVE NR	22.6% ( *n* = 7)	NRVT	3.2% ( *n* = 1)	49 years total cohort NR Complex aortic root	Mean F-up 5 years total cohort Complete F-up, 100%	NR
Knosalla, 9 German Heart Institute, Germany	2000	H = 47	mAVR = 15, tAVR = 3	1998–1996	All complex 27.7% PVE ( *n* = 18)	12.3% ( *n* = 8)	6.2% ( *n* = 4)	10.8% ( *n* = 7)	50.7 years	Range 3–12 years	86.2% ( *n* = 56)
Leung, 30 Prince Henry Hospital, Australia	1994	H = 2	AVR = 8	1989–1993	All complex 50% PVE ( *n* = 5)	40.0% ( *n* = 4)	10.0% ( *n* = 1)	10% ( *n* = 1)	45.8 years	Mean 21 months Range 4–46 months Complete F-up, 100%	100% ( *n* = 10)

Abbreviations: AVC, aortic valved conduit graft (tissue vs. mechanical not specified); AVR, aortic valve replacement; F-up, follow-up; H, homograft; mAVC, mechanical aortic valved conduit; mAVR, mechanical aortic valve replacement; NR, not reported; NRVT, not reported by valve type; PVE, prosthetic valve endocarditis; ReInf, reinfection; Reop, reoperation (repeat aortic or valvular intervention); SC, subcohort (where complex aortic infection was reported as a subset of a larger cohort study); tAVC, tissue aortic valved conduit; tAVR, tissue aortic valve replacement.

All of the included articles were retrospective observational studies comparing homograft performance to other forms of aortic valve/valved conduit graft replacement. The methodological assessment of the included studies using the Newcastle–Ottawa Quality Assessment Scale for cohort studies is shown in Table 3 . All 11 included studies were calculated as of moderate to good quality using this assessment scale.

**Table 3:** Newcastle–Ottawa Score for assessment of quality of cohort studies

Author (Year)	Selection	Comparability	Outcome	Total score
Elgalad (2019)	****	**	**	8*
Ramos (2016)	***	−	**	5*
Lee (2014)	****	**	***	9*
Jassar (2012)	****	**	***	9*
Avierinos (2007)	****	**	***	9*
David (2007)	***	−	***	6*
Leyh (2004)	**	−	***	5*
Siniawski (2003)	***	**	**	7*
Gulbins (2002)	***	*	***	7*
Knosalla (2000)	***	**	**	7*
Leung (1994)	***	**	***	7*

Note: The Newcastle-Ottawa Scale is a quality assessment tool that scores up to a maximum of 9 points over three different sections of criteria. Each ‘*’ in the table above indicates the number of points scored by each included study, under each heading.

### Patient Characteristics

In this meta-analysis 62.0% of patients were male, with a weighted mean age in those calculable for complex disease of 57.1 years. Of the 810 included patients, 47.0% had prosthetic valve infection with complex features. Streptococcal species and *Staphylococcal aureus* infections accounted for half of all episodes of complex infective endocarditis (as shown in Table 4 ). The nature of complex infection (abscess, pseudoaneurysm, etc.) and what shaped decision to undertake a particular style of root replacement was poorly described between studies on a valve implant basis; abscesses were most predominant, and surgeon preference seems to have shaped almost all decision around graft replacement (though some series commented on suitable homografts not being available at particular points as limiting their operative choice).

**Table 4:** Percent of complex episodes accounted for by various infectious agents

Organism	Percentage of cases
Streptococcus species	28.5
*Staphylococcus aureus*	21.5
Coagulase negative staphylococcus	12.3
Enterococcus	11.9
Culture negative	10.3
Fungal	3.1
Other	12.4

Several of the included studies did not differentiate between their valved conduits, and isolated valve replacements (without aortic conduit) with patch repairs, bioglue, or direct closure of abscesses. This is accounted for in the meta-analysis as results were analyzed by homografts versus all prosthesis types (valves and valved conduits) with subgroup analyses performed where sufficient detail was given (i.e., homografts vs. tissue valves or homografts vs. valved conduit grafts).

### Mortality

**Fig. 2:**
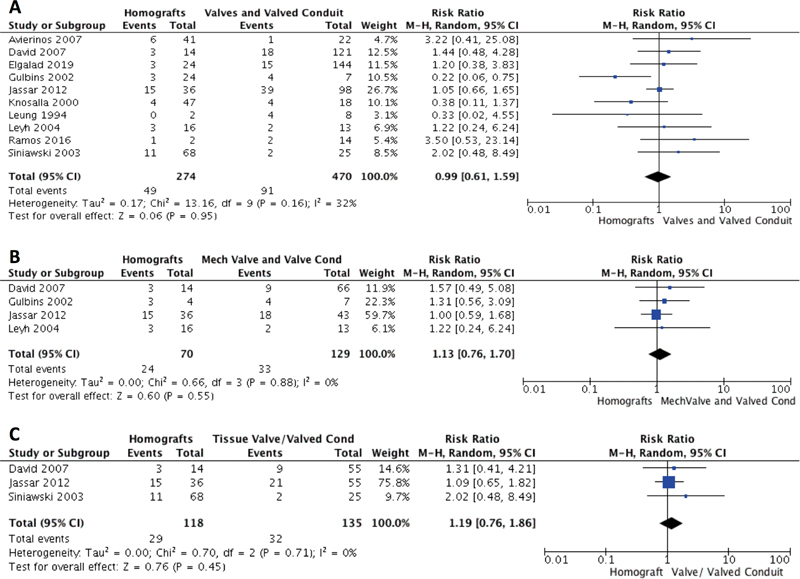
Total mortality rates of ( **A** ) homograft vs. all valves and valved conduits, ( **B** ) homograft vs. mechanical valves and mechanical valved conduits, and ( **C** ) homograft versus tissue valves and tissue valved conduits.

There were 175 (21.6%) deaths within the studies collated for meta-analysis with both aortic valve and valved conduit replacement. There was no statistically significant difference for total mortality between homografts and all valves/valved conduits ( Fig. 2A ) (RR 0.99; 95% CI, 0.61–1.59; *p* = 0.95; *I* ^2^ = 32%), homografts and mechanical valves and valved conduits ( Fig. 2B ) (RR 1.13; 95% CI, 0.76–1.70; *p* = 0.55; *I* ^2^ = 0%), or between homografts and tissue valves and valved conduits ( Fig. 2C ) (RR 1.19; 95% CI, 0.76–1.86; *p* = 0.45; *I* ^2^ = 0%). No statistically significant difference was observed in rates of total mortality between homografts and all valved conduit grafts (RR 1.19; 95% CI, 0.79–1.79, *p* = 0.41; *I* ^2^ = 0%) ( Supplementary Fig. S1 ). The majority of studies failed to report 5- and 10-year mortality. Total mortality was a heterogeneous group of endpoints based on mortality at the end of the study periods. There was no statistically significant difference in inpatient mortality between homografts and all valves/valved conduits ( Supplementary Fig. S2A ) (RR 0.99; 95% CI, 0.58–1.68, *p* = 0.96; *I* ^2^ = 37%), homografts and isolated tissue valves ( Supplementary Fig. S2B ) (RR 1.23; 95% CI, 0.70–2.19, *p* = 0.47; *I* ^2^ = 0%), or between homografts and all valved conduits (RR 1.41; 95% CI, 0.81–2.43, *p* = 0.22; I ^2^ = 0%) ( Supplementary Fig. S2C ).

### Reoperation

**Fig. 3:**
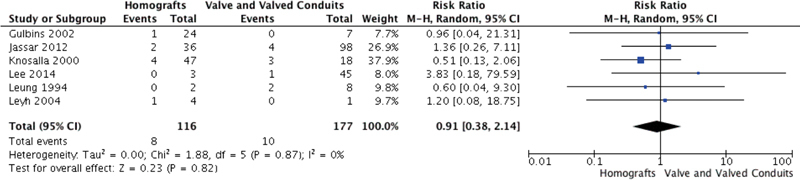
Rate of reoperation of homografts versus all valves and valved conduits.

Seven studies reported reoperation rates, with a low event rate of only 17 reoperations (4.5% at end of study follow-up for studies reporting reoperation). There was no significant difference in reoperation rates between homografts and all valves/valved conduit grafts (RR 0.91; 95% CI, 0.38–2.14, *p* = 0.82; *I* ^2^ = 0%) ( Fig. 3 ). There was also no difference in reoperation between homografts and tissue valves/valved conduit grafts (RR 1.14; 95% CI, 0.27–4.76, *p* = 0.85; *I* ^2^ = 0%) ( Supplementary Fig. S3 ).

### Reinfection

**Fig. 4:**
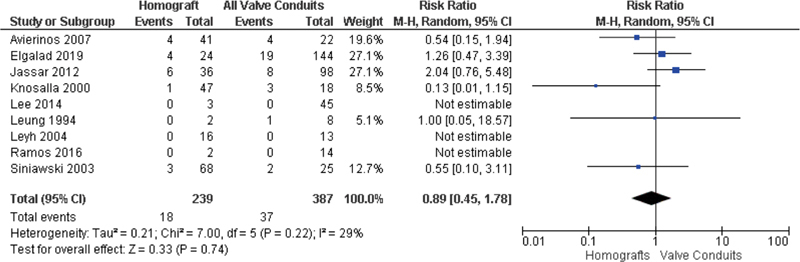
Rate of reinfection of homografts versus all valves and valved conduits.

A majority (9) of the included studies reported reinfection rates by valve type, with a total of 55 (8.8%) reinfections. There was no significant difference in reinfection rates between homografts and all valves/valved conduits (RR 0.89; 95% CI, 0.45–1.78; *p* = 0.74; *I* ^2^ = 29%) ( Fig. 4 ). It was inconsistently reported if these were the same as the initial infectious organism, or a second infectious agent. Forest plots of reinfection by valve type and time are available in Supplementary Fig. S4 . There was no difference between reinfection rates at the end of studies between homografts and tissue valves/valved conduit grafts (RR 1.43; 95% CI, 0.27–7.57; *p* = 0.68; *I* ^2^ = 58%) ( Supplementary Fig. S4A ), or 1-year reinfection rates between homografts and all valves/valved conduit grafts (RR 0.60; 95% CI, 0.19–1.85; *p* = 0.37; *I* ^2^ = 28%) ( Supplementary Fig. S4B ). Reinfection rates at 5-year follow-up between homografts and all valves/valved conduit grafts were also not statistically significant (RR 0.86; 95% CI, 0.25–2.99, *p* = 0.82; *I* ^2^ = 52%) ( Supplementary Fig. S4C ). No significant difference was also observed between homografts and all valved conduit grafts at the end of reported follow-up (RR 0.94; 95% CI, 0.35–2.55; *p* = 0.91; *I* ^2^ = 44%) ( Supplementary Fig. S5 ).

## Discussion

The results of this meta-analysis revealed no statistically significant difference in rates of overall mortality, reinfection, or reoperation between homografts and valve replacement/valved conduit grafts for complex aortic valve endocarditis used at the discretion of the operative surgeon. This indeed reflects that the choice of conduit is more complex than simple dogma, and depends upon patient characteristics, technical considerations, degree of tissue to be replaced, surgeon skill and preferences, as well as availability of various conduit types.

Due to the high-risk nature of these patients, the need for urgent intervention, the often frequent intraoperative change in clinical findings, and the very heterogeneous nature of patients with complex aortic valve endocarditis it would be difficult to coordinate these large multicenter randomized controlled trials necessary to appropriately investigate this. Kim and Sundt, 11 estimated that there would need to be 3,000 patients enrolled to demonstrate (with adequate power) the hypothesis that homografts are better than biologic or mechanical prostheses.

Furthermore, the largest volume retrospective studies of large databases, such as Savage et al, 31 examining the STS database, have been limited by these databases previously not capturing data around aortic root abscesses. This has now been addressed and should be focused on by future analyses from these databases. Additionally, for future trials, there is a lack of consensus nature of reporting subtypes of complex aortic valve endocarditis with aortic root involvement and this needs to be addressed to ensure crucial future research can be completed in a less heterogeneous manner to provide higher quality evidence to shape future treatment guidelines.

As described above, there could be several underappreciated factors that contributed to the findings in this current study. However, these findings are similar to those of Yanagawa et al, 32 in a meta-analysis of homograft versus conventional prosthesis for management of general aortic valve endocarditis, and Perrotta and Lentini, 16 in patients with severe aortic valve endocarditis comparing stentless valves to homografts, both of which found comparable rates of mid-to-long term survival between homografts and different prosthesis types.

In the current meta-analysis, there was a low rate of reinfection with homografts and all valve prostheses. These findings echo those of Yanagawa et al 32 and Perrotta and Lentini, 16 in patients with severe aortic valve endocarditis, who both found comparable rates of reinfection between the two groups. These collective findings speak generally against the typical surgical dogma of homografts, having less prosthetic tissue to harbor residual/recurrent infection, and supports those suggestions by Feindel, 33 David et al, 8 and others that it is of equal if not greater importance to ensure adequate debridement of all infected tissue as an underlying principle of avoiding reinfection.

We found no statistically significant long-term difference in rates of reoperation. This is similar to the findings of Yanagawa et al 32 and Perrotta and Lentini, 16 for overall prostheses and stentless conduits. However, our finding of rates of reoperation for homografts versus mechanical valves contrasts with Yanagawa et al, 32 suggestion that homografts may be associated with greater risk of reoperation compared with mechanical valves. Though we did see an increased rate of reoperation in homografts rather than mechanical valves (4.8% vs. 1.7% at 5 years) this did not reach statistical significance (RR 2.30; 95% CI, 0.35–15.05). This was with a limited number ( *n* = 180) of patients and only at 5 years' follow-up. It would not be surprising if with greater time further degrees of homograft degeneration were noted in keeping with the results of Flameng et al, 17 who in a series of homografts for infectious endocarditis saw a 40% structural valve degeneration at 10 years. Therefore, further assessment with larger volume studies and longer-term follow-up is warranted to better quantify the risk of reoperation in homografts compared with other valves and valved conduits.

When valved aortic graft conduits were compared with homografts the results for mortality, reinfection, and reoperation were all grossly similar. Again, this may represent that the Dacron involved in aortic grafting and the prosthetic valves do not offer a surface more likely to retain infectious organisms when thorough debridement has been performed in vivo. Alternatively, it may represent cases in which there was less infectious burden originally and less anatomical resection needing to be performed. However, based on the studies available, the performance of valved conduit grafts was not inferior to that of homografts with regards to mortality, reoperation, and reinfection. It would seem that if complete debridement of infection is undertaken, numerous valve and valved conduit types can be safely used to reconstruct the aortic root without increased rates of reinfection. However, particularly given the limited number of patients, and the previous assessment that up to 3,000 patients would be required to assess whether valved conduits are superior to homografts, it may be that these results represent a Type II error. Neither this meta-analysis nor the retrospective cohorts that comprise it were able to answer this, and it would be dangerous to conclude noninferiority on this basis alone.

Despite the recent trend toward increasing use of conventional valve and valved conduits in complex aortic valve endocarditis within the literature, there has also been increasing access to homografts for infective endocarditis. In a study examining homograft use via the Belgian and European Homograft registry it was reported that 137% more homografts were used in the period from 2000 to 2010 compared with 1990 to 2000. 34

### Limitations

Many of the limitations faced in this meta-analysis have been highlighted above in the discussion and echo those raised by the authors of many of the included studies. The population involved is highly heterogeneous within studies, ranging from those with only a small abscess cavity easily primarily closed, to highly moribund patients with gross aorto-ventricular discontinuity undergoing salvage surgery. Although some studies had high rates of follow-up (some complete follow-up), others had greater than 20% loss to follow-up, affecting the reliability of the results. All the included studies were also retrospective in nature, with no prospective randomized control trials available. This obviously affects the ability to compare readily between the homograft and valve/valved conduit group as extent of infection and nature of anatomical replacement likely shaped the operation patients received. Indeed, as highlighted above, we lack a clear, universal system for classifying complex aortic valve endocarditis, and this would be of value to help better classify and compare future research efforts.

## Conclusions

We found similar outcomes with respect to mortality, freedom from reoperation, and freedom from reinfection between homografts and valve replacement/valved conduits. This contradicts previously held dogma that homograft use should decrease rates of reinfection in infective endocarditis. It would seem that if appropriate resection and debridement of infected tissue can be achieved, valve replacement can be safely achieved with the surgeon's preferred conventional valve, valved conduit graft, or homograft depending upon what the anatomical deficit requires. However, these findings may very well represent a Type II error. Several factors related to the combined studies limit the ability of this meta-analysis to reach further definitive conclusions, particularly in relation to the difficulty in further defining the anatomical requirements of these patients. Overall, there is a lack of high-quality comparative studies, and this complex and controversial area would benefit from the assessment of large national/multinational databases better defining complex aortic valve infective endocarditis, or large volume multicenter randomized controlled trials, though we acknowledge these will be difficult to perform in this high-risk, heterogeneous group of patients.
