# Supplementary material for: Homograft Versus Valves and Valved Conduits for Extensive Aortic Valve Endocarditis with Aortic Root Involvement/Destruction: A Systematic Review and Meta-Analysis
Source: Aorta (Stamford). 2022 Aug 7;10(2):43–51. doi: 10.1055/s-0042-1743110 (PMC9357462; doi:10.1055/s-0042-1743110)
Supplement: Supplementary file 1 — Supplementary Material [file 10-1055-s-0042-1743110-s200075.pdf]

Study or Subgroup	Homografts Events	Homografts Total	Aortic Composites Events	Aortic Composites Total	Weight	Risk Ratio M-H, Random, 95% CI
Elgalad 2019	3	24	0	9	2.0%	2.80 [0.16, 49.43]
Jassar 2012	15	36	39	98	80.4%	1.05 [0.66, 1.65]
Leyh 2004	3	16	2	13	6.3%	1.22 [0.24, 6.24]
Ramos 2016	1	2	1	8	3.2%	4.00 [0.40, 39.83]
Siniawski 2003	11	68	2	25	8.1%	2.02 [0.48, 8.49]
**Total (95% CI)**		**146**		**153**	**100.0%**	**1.19 [0.79, 1.79]**
Total events	33		44			

Heterogeneity: $Tau^2 = 0.00; Chi^2 = 2.31, df = 4 (P = 0.68); I^2 = 0\%$
Test for overall effect: Z = 0.82 (P = 0.41)

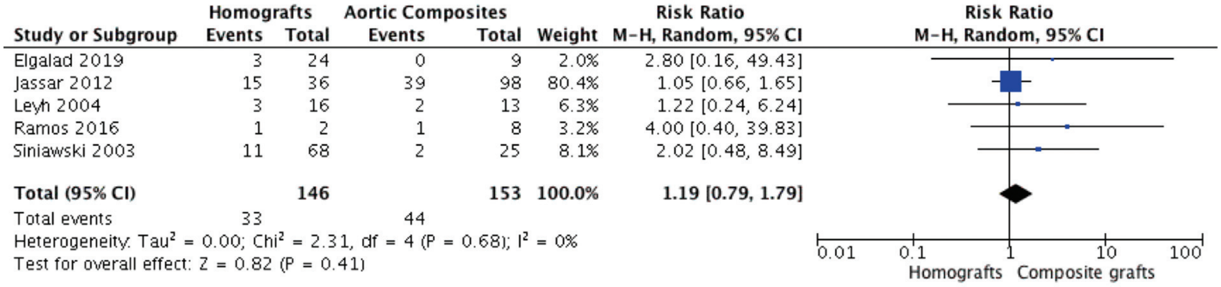

Supplementary Fig. S1 Rates of total mortality of homografts versus all valved conduit grafts.

A

Study or Subgroup	Homografts Events	Homografts Total	Valve and Valved Conduits Events	Valve and Valved Conduits Total	Weight	Risk Ratio M-H, Random, 95% CI
Avierinos 2007	6	41	1	22	5.5%	3.22 [0.41, 25.08]
David 2007	3	14	18	121	13.1%	1.44 [0.48, 4.28]
Elgalad 2019	3	24	15	144	12.2%	1.20 [0.38, 3.83]
Gulbins 2002	3	24	4	7	11.3%	0.22 [0.06, 0.75]
Jassar 2012	9	36	21	98	19.7%	1.17 [0.59, 2.31]
Knosalla 2000	4	47	4	15	11.1%	0.32 [0.09, 1.12]
Leung 1994	0	2	4	8	3.6%	0.33 [0.02, 4.55]
Leyh 2004	3	16	2	13	7.8%	1.22 [0.24, 6.24]
Ramos 2016	1	2	2	14	6.3%	3.50 [0.53, 23.14]
Siniawski 2003	11	68	2	25	9.4%	2.02 [0.48, 8.49]
**Total (95% CI)**		**274**		**467**	**100.0%**	**0.99 [0.58, 1.68]**
Total events	43		73			

Heterogeneity: $Tau^2 = 0.25; Chi^2 = 14.33, df = 9 (P = 0.11); I^2 = 37\%$
Test for overall effect: Z = 0.05 (P = 0.96)

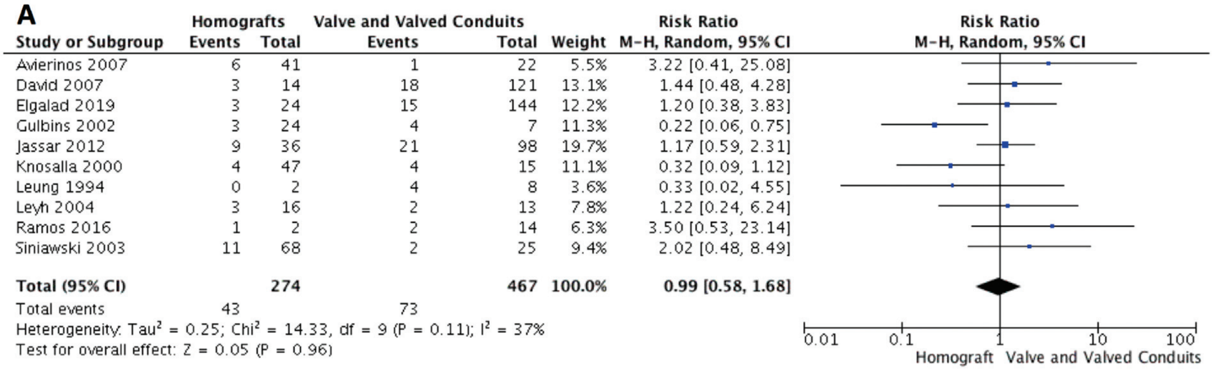

B

Study or Subgroup	Homografts Events	Homografts Total	Tis Valve and Valved Cond Events	Tis Valve and Valved Cond Total	Weight	Risk Ratio M-H, Random, 95% CI
David 2007	3	14	9	55	24.0%	1.31 [0.41, 4.21]
Jassar 2012	9	36	13	55	60.0%	1.06 [0.51, 2.21]
Siniawski 2003	11	68	2	25	15.9%	2.02 [0.48, 8.49]
**Total (95% CI)**		**118**		**135**	**100.0%**	**1.23 [0.70, 2.19]**
Total events	23		24			

Heterogeneity: $Tau^2 = 0.00; Chi^2 = 0.65, df = 2 (P = 0.72); I^2 = 0\%$
Test for overall effect: Z = 0.72 (P = 0.47)

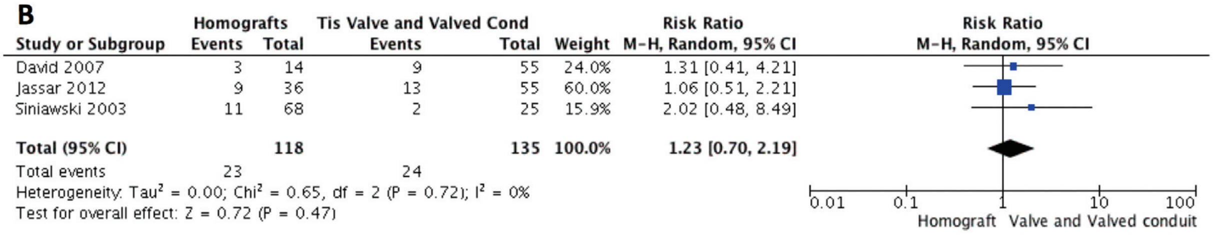

C

Study or Subgroup	Homografts Events	Homografts Total	Aortic Composites Events	Aortic Composites Total	Weight	Risk Ratio M-H, Random, 95% CI
Elgalad 2019	3	24	0	9	3.6%	2.80 [0.16, 49.43]
Jassar 2012	9	36	21	98	64.8%	1.17 [0.59, 2.31]
Leyh 2004	3	16	2	13	11.3%	1.22 [0.24, 6.24]
Ramos 2016	1	2	1	8	5.7%	4.00 [0.40, 39.83]
Siniawski 2003	11	68	2	25	14.6%	2.02 [0.48, 8.49]
**Total (95% CI)**		**146**		**153**	**100.0%**	**1.41 [0.81, 2.43]**
Total events	27		26			

Heterogeneity: $Tau^2 = 0.00; Chi^2 = 1.59, df = 4 (P = 0.81); I^2 = 0\%$
Test for overall effect: Z = 1.22 (P = 0.22)

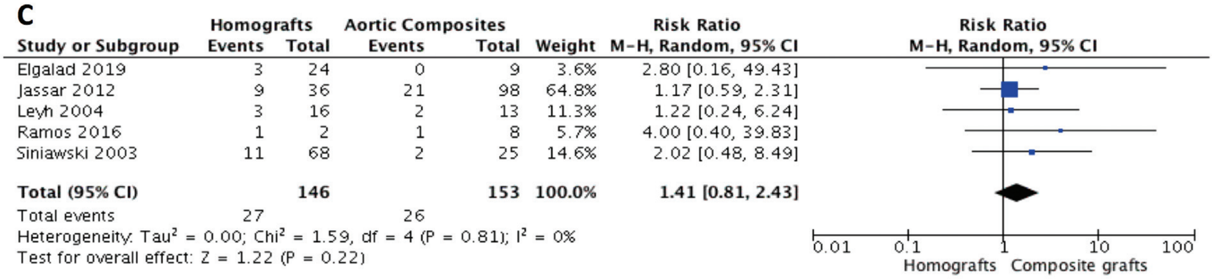

Supplementary Fig. S2 Rates of inpatient mortality for (**A**) homografts versus all valves and valved conduits, (**B**) homografts versus tissue valves, and (**C**) homografts versus all valved conduit grafts.

Study or Subgroup	Homografts Events	Homografts Total	Tiss Valve and Valved Con Events	Tiss Valve and Valved Con Total	Weight	Risk Ratio M-H, Random, 95% CI
Gulbins 2002	1	24	0	7	21.2%	0.96 [0.04, 21.31]
Jassar 2012	2	36	2	55	55.6%	1.53 [0.23, 10.36]
Lee 2014	0	3	1	7	23.2%	0.67 [0.03, 12.96]
**Total (95% CI)**		**63**		**69**	**100.0%**	**1.14 [0.27, 4.76]**
Total events	3		3			

Heterogeneity: $Tau^2 = 0.00; Chi^2 = 0.23, df = 2 (P = 0.89); I^2 = 0\%$
Test for overall effect: Z = 0.18 (P = 0.85)

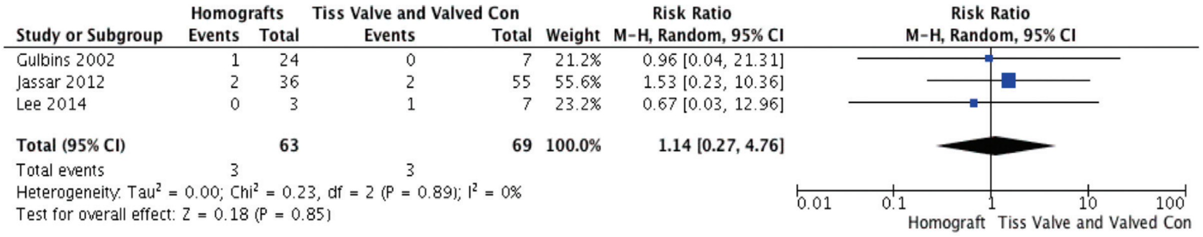

Supplementary Fig. S3 Rates of reoperation of homografts versus tissue valves and tissue valved conduits.

 © 2022. The Author(s).

A

Study or Subgroup	Homografts Events	Homografts Total	Tissue Valve Conduits Events	Tissue Valve Conduits Total	Weight	Risk Ratio M-H, Random, 95% CI
Jassar 2012	6	36	3	55	55.5%	3.06 [0.82, 11.45]
Lee 2014	0	3	0	7		Not estimable
Siniawski 2003	3	68	2	25	44.5%	0.55 [0.10, 3.11]
**Total (95% CI)**		**107**		**87**	**100.0%**	**1.43 [0.27, 7.57]**
Total events	9		5			

Heterogeneity: Tau² = 0.85; Chi² = 2.38, df = 1 (P = 0.12); I² = 58%
Test for overall effect: Z = 0.42 (P = 0.68)

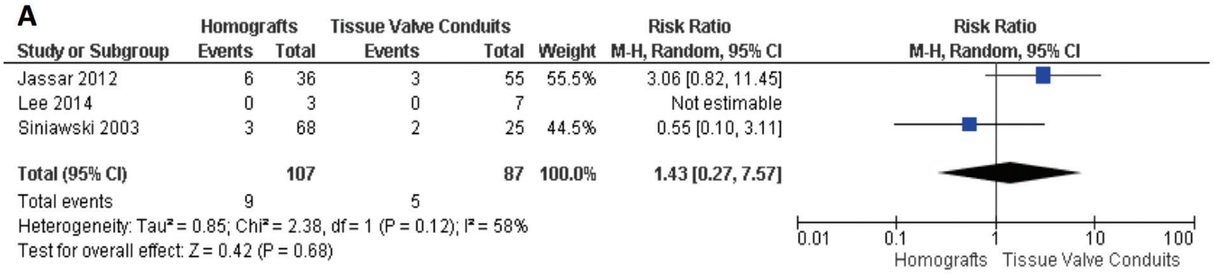

B

Study or Subgroup	Homografts Events	Homografts Total	Valve Conduits Events	Valve Conduits Total	Weight	Risk Ratio M-H, Random, 95% CI
Avierinos 2007	4	41	4	22	46.5%	0.54 [0.15, 1.94]
Elgalad 2019	2	17	10	106	40.7%	1.25 [0.30, 5.21]
Knosalla 2000	0	45	2	18	12.8%	0.08 [0.00, 1.64]
Lee 2014	0	3	0	45		Not estimable
Ramos 2016	0	2	0	14		Not estimable
**Total (95% CI)**		**108**		**205**	**100.0%**	**0.60 [0.19, 1.85]**
Total events	6		16			

Heterogeneity: Tau² = 0.29; Chi² = 2.77, df = 2 (P = 0.25); I² = 28%
Test for overall effect: Z = 0.90 (P = 0.37)

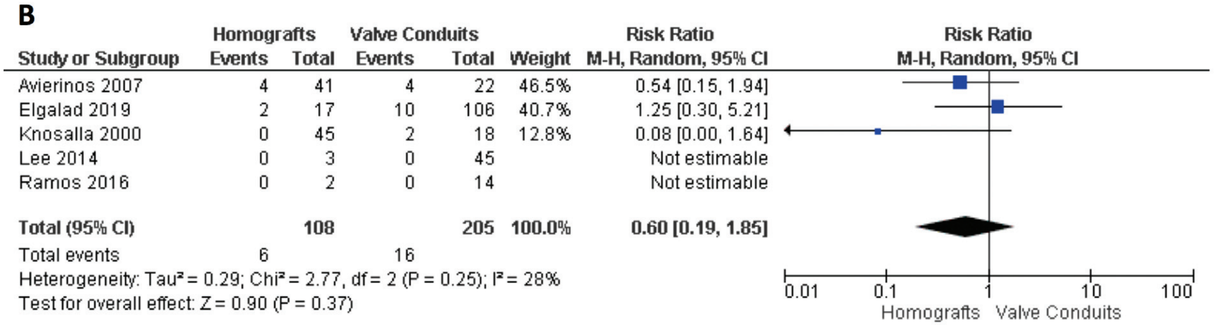

C

Study or Subgroup	Homografts Events	Homografts Total	Valve Conduits Events	Valve Conduits Total	Weight	Risk Ratio M-H, Random, 95% CI
Elgalad 2019	2	17	9	50	34.7%	0.65 [0.16, 2.73]
Jassar 2012	6	36	8	98	45.7%	2.04 [0.76, 5.48]
Knosalla 2000	1	47	2	18	19.6%	0.19 [0.02, 1.98]
Lee 2014	0	3	0	45		Not estimable
**Total (95% CI)**		**103**		**211**	**100.0%**	**0.86 [0.25, 2.99]**
Total events	9		19			

Heterogeneity: Tau² = 0.62; Chi² = 4.20, df = 2 (P = 0.12); I² = 52%
Test for overall effect: Z = 0.23 (P = 0.82)

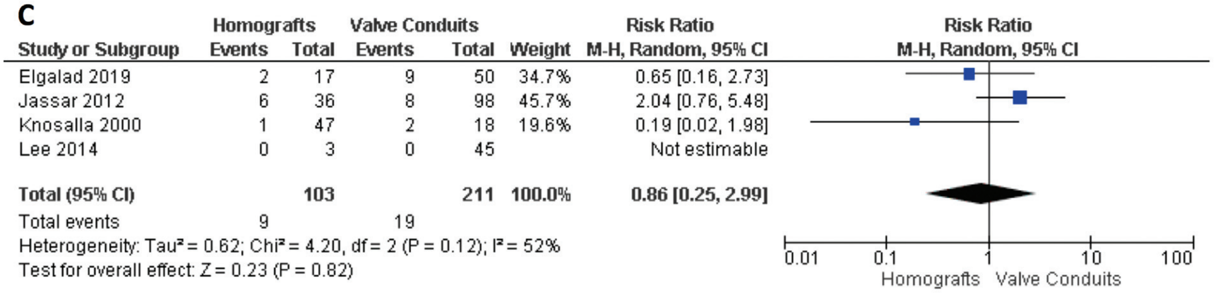

Supplementary Fig. S4 Rates of reinfection of (**A**) homografts versus tissue valves and valved conduits at end of study follow-up, (**B**) homografts versus all valves and valved conduits at 1-year, and (**C**) homografts versus all valves and valved conduits at 5 years.

Study or Subgroup	Homografts Events	Homografts Total	Aortic Composites Events	Aortic Composites Total	Weight	Risk Ratio M–H, Random, 95% CI
Elgalad 2019	4	24	3	9	33.5%	0.50 [0.14, 1.81]
Jassar 2012	6	36	8	98	43.4%	2.04 [0.76, 5.48]
Leyh 2004	0	16	0	13		Not estimable
Ramos 2016	0	2	0	8		Not estimable
Siniawski 2003	3	68	2	25	23.1%	0.55 [0.10, 3.11]
**Total (95% CI)**		**146**		**153**	**100.0%**	**0.94 [0.35, 2.55]**
Total events	13		13			

Heterogeneity: Tau² = 0.34; Chi² = 3.56, df = 2 (P = 0.17); I² = 44%
Test for overall effect: Z = 0.12 (P = 0.91)

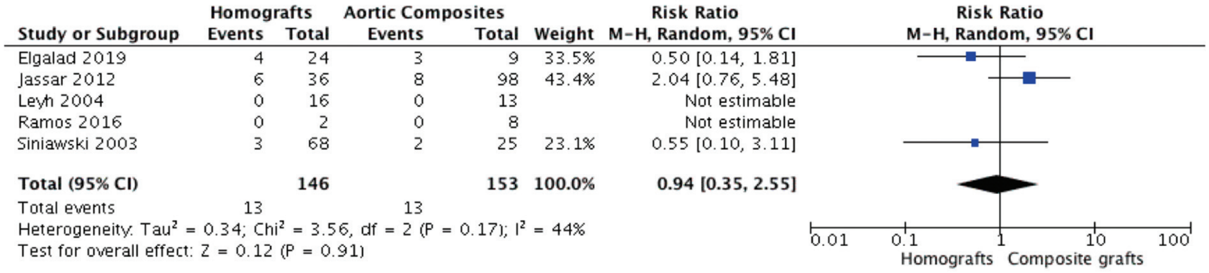

Supplementary Fig. S5 Rates of reinfection of homografts versus all valved conduit grafts.

 © 2022. The Author(s).
